# Long Non-Coding RNA *Lacuna* Regulates Neuronal Differentiation of Neural Stem Cells During Brain Development

**DOI:** 10.3389/fcell.2021.726857

**Published:** 2021-11-24

**Authors:** Elpinickie Ninou, Artemis Michail, Panagiotis K. Politis

**Affiliations:** ^1^ Center for Basic Research, Biomedical Research Foundation of the Academy of Athens, Athens, Greece; ^2^ Department of Biology, National and Kapodistrian University of Athens, Athens, Greece; ^3^ Department of Biology, University of Patras, Patras, Greece

**Keywords:** Tbr2/Eomes, NONMMUT071331, non-coding genome, lncRNAs, KRAB/CRISPR/dCas9

## Abstract

Although long non-coding RNAs (lncRNAs) is one of the most abundant classes of RNAs encoded within the mammalian genome and are highly expressed in the adult brain, they remain poorly characterized and their roles in the brain development are not well understood. Here we identify the lncRNA *Lacuna* (also catalogued as NONMMUT071331.2 in NONCODE database) as a negative regulator of neuronal differentiation in the neural stem/progenitor cells (NSCs) during mouse brain development. In particular, we show that *Lacuna* is transcribed from a genomic locus near to the Tbr2/Eomes gene, a key player in the transition of intermediate progenitor cells towards the induction of neuronal differentiation. *Lacuna* RNA expression peaks at the developmental time window between E14.5 and E16.5, consistent with a role in neural differentiation. Overexpression experiments in *ex vivo* cultured NSCs from murine cortex suggest that *Lacuna* is sufficient to inhibit neuronal differentiation, induce the number of Nestin+ and Olig2+ cells, without affecting proliferation or apoptosis of NSCs. CRISPR/dCas9-KRAB mediated knockdown of *Lacuna* gene expression leads to the opposite phenotype by inducing neuronal differentiation and suppressing Nestin+ and Olig2+ cells, again without any effect on proliferation or apoptosis of NSCs. Interestingly, despite the negative action of *Lacuna* on neurogenesis, its knockdown inhibits Eomes transcription, implying a simultaneous, but opposite, role in facilitating the Eomes gene expression. Collectively, our observations indicate a critical function of *Lacuna* in the gene regulation networks that fine tune the neuronal differentiation in the mammalian NSCs.

## Introduction

Understanding the molecular mechanisms that control the mammalian brain development is one of the most challenging goals of biomedical sciences. For a long time, it was thought that an intricate network of transcription factors and chromatin modulators is responsible for activating or repressing specific genes or gene circuitries to control proliferation, differentiation and specification of neural stem/progenitor cells (NSCs) during the brain formation (Corbin et al., 2008; Martynoga et al., 2012; Urban and Guillemot, 2014). However, the emergence of new genome sequencing technologies and the experimental data from large consortia such as ENCODE and FANTOM have radically changed our view of the organization, activity and regulation of the mammalian genome (Carninci et al., 2005; Katayama et al., 2005; Birney et al., 2007; Consortium, 2012). It has now become clear that most of the genome is transcribed and produces a large number of regulatory RNA molecules that were not previously known. Among them, long non-coding RNAs (lncRNAs) are transcripts larger than 200 nt that can be modified by 5′-capping, polyadenylation and splicing, similar to mRNAs, yet they are not translated into proteins (Maeda et al., 2006; Djebali et al., 2012). Their genomic location varies as they can be found in introns of protein coding genes, sense or anti-sense to other genes, intergenic regions (Kapranov et al., 2007; Seila et al., 2008), promoters (Hung et al., 2011), enhancers (Ørom et al., 2010), gene regulatory regions like UTRs (Mercer et al., 2011), even telomeres (Azzalin et al., 2007). Most importantly, lncRNAs appear to be involved in the regulatory networks that control stem cell pluripotency, carcinogenesis, growth, and function of many tissues and organs (Mercer et al., 2009; Sheik Mohamed et al., 2010; Guttman et al., 2011; Ng et al., 2012; Antoniou et al., 2014; Ramos et al., 2015; Giakountis et al., 2016; Zarkou et al., 2018; Chi et al., 2019; Malissovas et al., 2019).

Likewise, many recent studies indicate that lncRNAs are involved in homeostasis and function of the mammalian brain as well as in the pathophysiology of brain related diseases, including neurodevelopmental disorders (Faghihi et al., 2008; Johnson, 2012; Barry et al., 2014; Briggs et al., 2015; Elkouris et al., 2019; Hosseini et al., 2019; Tsagakis et al., 2020). Accordingly, it has been reported that thousands of lncRNAs are expressed in the embryonic and adult mammalian brain in a highly patterned and specific manner (Ponjavic et al., 2009; Fatica and Bozzoni, 2014; Andersen and Lim, 2018), yet they remain poorly characterized and their roles in brain development have not been extensively studied. Towards this direction, it has been suggested that a significant proportion of lncRNAs may have the ability to regulate *in cis* the neighboring protein-coding genes *via* the reorganization of chromatin microenvironment (Bond et al., 2009; Zhang et al., 2012; Sauvageau et al., 2013). Along these lines, we have previously proposed that a subset of lncRNAs that are transcribed in close genomic proximity to genes encoding for transcription factors with critical roles in the brain development, may also be able to regulate these genes and therefore be involved in neural development (Antoniou et al., 2014).

Here we identify *Lacuna* (also catalogued as TCONS_00034309 or NONMMUT071331.2 in NONCODE database) as a lncRNA gene localized near to the Eomes transcription factor gene, and that *Lacuna* is highly and differentially expressed during embryonic development of mouse cortex. Interestingly, *Lacuna* has not been previously studied in the context of nervous system or any other tissue, organ or cell type. By gain- and loss-of-function experiments in NSCs isolated from the murine embryonic cortex, we show that *Lacuna* suppresses neuronal differentiation, possibly *via* an *in trans* action. At the same time, *Lacuna* expression is required for the Eomes gene expression, a function that is opposite to its negative role in neurogenesis, since Eomes promotes neuronal differentiation (Englund et al., 2005; Arnold et al., 2008b; Mihalas et al., 2016; Sessa et al., 2017; Hevner, 2019; Lv et al., 2019). These two opposite functions may indicate that *Lacuna* is an interconnecting node in the gene regulatory networks that fine tune the differentiation in NSCs during development.

## Materials and Methods

### Ethics Statement

The study protocol took place in the animal facilities of the Center for Experimental Surgery of the Biomedical Research Foundation of the Academy of Athens. All animals were handled in strict accordance with good animal practice as defined by the relevant European and Greek animal welfare bodies.

### Culture of NSCs, Overexpression and Knockdown Studies

Neurosphere cultures from E14.5 mouse cortical tissue were prepared as previously described (Politis et al., 2008; Kaltezioti et al., 2010; Kaltezioti et al., 2014; Stergiopoulos and Politis, 2016). Proliferation or differentiation assays were performed after dissociation of NSCs to single cells, plating onto poly-L-lysine (Sigma) coated coverslips in 24-well plates at a density of 1 × 10^5^ and further *ex vivo* culture for 2 or 3 days with or without Growth Factors, respectively, in a 37°C humidified incubator with 5% CO_2_. The cells were maintained in suspension in full medium with growth factors as follows: 1:1 mixture of Dulbecco’s modified Eagle’s medium (1 g/L d-glucose, l-glutamine, pyruvate; Sigma), F-12 nutrient mixture (Sigma) plus 20 ng/ml human epidermal growth factor (EGF; R&D Systems) and 20 ng/ml human basic fibroblast growth factor (R&D Systems), 20 μg/ml insulin (Sigma), 1x B27 supplement (Gibco), 0.25 mM L-glutamine, and 1% penicillin/streptomycin to promote the production of floating neurospheres. The neurospheres were then passaged 3–4 times before the assays. Differentiated neurosphere cultures were maintained in minus growth factors conditions, the same as the full medium plus growth factors without human EGF and basic FGF, in order to promote differentiation.

For overexpression studies, the *Lacuna* lncRNA sequence was first cloned into pcDNA3.1 (GenScript) and then it was sub-cloned into pCAGGs vector. Together with pCAGGs-*Lacuna* a pCAGGs-GFP plasmid was used (in a ratio of 3:1) in order to visually mark the transfected cells. Empty pCAGGs together with pCAGGs-GFP (in a ratio of 3:1) were also used as a control for the overexpression experiments.

For knockdown studies, a CRISPR-dCas9-KRAB effector system was used, kindly provided by Dr. Pantelis Hatzis (BSRC, Al Fleming, Athens Greece). This system consists of two plasmids (1:1): a pHR-KRAB-dCas9-mCherry and a pU6-sgRNA-EF1Alpha-puro-T2A-BFP (without gRNA for control and with gRNAs against *Lacuna* sequence for *Lacuna* knockdown). gRNAs were designed using the GenCRISPR gRNA Design Tool (https://www.genscript.com/gencrispr-grna-design-tool.html?src=google&gclid=CjwKCAjwn6GGBhADEiwAruUcKty8qKnSWhOxCpac_VrRqHDGm4a7RgBDp01gPjihJLS0Ydvtzw482BoC7WMQAvD_BwE) to target the first exon of *Lacuna* sequence.

NSCs were transfected using an AMAXA electroporator (Lonza) with 6 μg of total plasmid DNA per electroporation, according to manufacturer’s instructions, as also previously described (Kaltezioti et al., 2010; Kaltezioti et al., 2014). After electroporation, NSCs were incubated overnight in full medium with 1% FBS in order for them to surpass electroporation shock and then they were incubated according to the experiment.

In general, NSCs cultures exhibit a remarkable heterogeneity and variability that become larger, when they are under differentiation conditions. This variability depends on many factors, e.g., the initial material, isolation conditions of embryonic cortices and brains or even the state of the mother, the dissociation of the tissue, the growth rate of the culture, the efficiency and the cell death induced by the Amaxa electroporation system, as well as factors related to the attachment material. For this reason, we performed the same experiments multiple times, and we always included the appropriate controls, so that most of the above factors would be the same between the samples and biological replicates.

### RNA Extraction and Real-Time RT-qPCR Analysis

Total RNA was isolated by cells and tissues with TRI reagent solution (AM9738, Ambion/RNA, Life Technologies) according to manufacturer’s instructions, followed by treatment with RQ1 DNase (Promega, Madison, WI, United States). RNA concentration and purity was measured by Nanodrop 2000c (Thermo), and 1.5 μg of RNA was used for cDNA synthesis using the SuperScript First-Strand Synthesis System (Invitrogen, Carlsbad, United States) together with random hexamer primers. Quantitative Real time RT-PCR analysis was performed in a LightCycler 96 Instrument (Roche). Measured values were normalized using beta actin or Gapdh and RPL13A mRNA levels as internal references.

Primers that were used for real-time RT-qPCR are presented in the above table.

**Table:** 

Genes	Sequence	
beta actin	Forward	CCCAGGCATTGCTGACAG
	Reverse	TGG​AAG​GTG​GAC​AGT​GAG​GC
Gapdh	Forward	TGC​CAC​TCA​GAA​GAC​TGT​GG
	Reverse	TTC​AGC​TCT​GGG​ATG​ACC​TT
RPL13A	Forward	ATG​ACA​AGA​AAA​AGC​GGA​TG
	Reverse	CTT​TTC​TGC​CTG​TTT​CCG​TA
Eomes	Forward	TTC​CGG​GAC​AAC​TAC​GAT​TCA
	Reverse	ACGCCGTACCGACCTCC
*Lacuna*	Forward	CGG​GTC​CTC​TCA​AGT​CAG​TC
	Reverse	GTT​GCT​TCC​ACA​TGC​TTC​CT
Golga4	Forward	GTT​GAA​GCA​CAC​GTC​CAC​AC
	Reverse	AGT​TCG​GCT​TCC​ACC​TCT​TG
Gm33450	Forward	GGA​GGA​CGG​GAA​AGA​CTG​TC
	Reverse	TTG​TTG​TAG​GGC​TGG​CTC​TG
U6long	Forward	GTG​CTC​GCT​TCG​GCA​GCA​CA
	Reverse	GGA​ACG​CTT​CAC​GAA​TTT​GCG​TGT​CAT
18s	Forward	TTGACGGAAGGGCACCAC
	Reverse	ACCACCACCCACGGAATC
7SK	Forward	TTC​CCC​GAA​TAG​AGG​AGG​AC
	Reverse	GCC​TCA​TTT​GGA​TGT​GTC​TG

The folds of expression were calculated using the 2^−ΔΔCT^ Method (Livak and Schmittgen, 2001).

### RT-PCR Mapping of *Lacuna* Transcript

As the *Lacuna* lncRNA is not yet annotated, we specified the boundaries of its three exons using appropriate primers and PCR (KAPA Taq PCR Kit). The templates were cDNAs derived from RNA of embryonic mouse cortices in embryonic days E12, E14, E16, E18, and newborns P0. Then, we performed gel electrophoresis of PCR products using the appropriate DNA ladder (Quick Load Purple 100 bp DNA Ladder, #NO551G, New England Biolabs).

Primers that were used for PCR are presented in the following list:

Forward Primers:
Primer-1: CTG​GCA​CTG​AGT​ACT​CTG​GGG​ACC​CAA​C
Primer-2: ACT​CTG​GGG​ACC​CAA​CTT​TT
Primer-3: CGG​GTC​CTC​TCA​AGT​CAG​TC
Primer-4: AAA​TCT​CCA​CCG​GGT​GAA​AG
Reverse Primers:
Primer-5: GTG​GGC​TTC​ATT​TCT​TCA​GC
Primer-6: GTT​GCT​TCC​ACA​TGC​TTC​CT
Primer-7: GTC​TAT​TTC​AAG​TCT​TGT​ATA​TTT​TTG​CAC​CG

### Subcellular Fractionation

Neurospheres were cultured in full medium plus growth factors and were harvested in passage 2. Subcellular fractionation was performed with PARIS Kit Protein and RNA isolation system (Ambion, AM 1921). Nuclear and cytoplasmic samples were obtained and then, we performed RNA isolation, cDNA synthesis and real time RT-qPCR analysis. Efficient fractionation of the subcellular compartments and normalization of the measured values were evaluated by using Gapdh, U6long, 18s, and 7sk primer pairs.

### Immunofluorescence

For the cell immunostaining experiments, primary cells were cultured onto poly-L-lysine (Sigma) coated coverslips in 24-well plates. In particular, after electroporation, NSCs were cultured for 24 h in full medium (+ GF) to recover from the electroporation reaction. Then, these cells were cultured either in the presence of growth factors for 48 h (to measure proliferation and associated markers), or in the absence of growth factors for 72 h (to measure differentiation and associated markers). At the end of the experiment, cells were fixed on the coverslips with 4% PFA. The coverslips were blocked with 5% FBS in 1x PBS, containing 0.3% triton X-100 for 2 h at room temperature (RT). Next, they were incubated with primary antibodies at 4°C overnight, followed by secondary antibodies for 2 h at RT. Finally, they were incubated with DAPI, diluted in 1X PBS for 10 min at RT, followed by mounting with MOWIOL. The primary antibodies in the immunofluorescence were anti-BrdU (Abcam, 6326) (1:400 dilution), rabbit anti-cleaved caspase 3 (Cell Signaling, 9661) (1:800 dilution), mouse Tuj1/anti-beta III tubulin (Covance, MMS-435P-250) (1:1,000 dilution), anti-GFAP (Abcam, 4674) (1:1,500 dilution), rabbit anti-TBR2 (Abcam, Ab23345) (1:1,000 dilution), goat anti-Olig2 (1:400 dilution), mouse anti-NeuN (Millipore, MAB337) (1:200 dilution), chicken anti-GFP (Abcam, Ab13790), chicken anti-mCherry (Novus, NBP2-25158) (1:1,000 dilution). The secondary antibodies were donkey anti-Rabbit 488 (AlexaFluor), donkey anti-Mouse 568 (AlexaFluor), donkey anti-Rabbit 647 (AlexaFluor), donkey anti-Chicken 488 (AlexaFluor), donkey anti-Rat 488 (AlexaFluor).

### *In Situ* Hybridization on Cryosections

Mouse embryonic brains of various developmental stages were incubated in 4% PFA for 4 h and left overnight in 30% sucrose in PBS for cryoprotection. Then, the tissue was embedded in OCT, sectioned transversely at 12 μm and collected on super-frost slides. Non-radioactive *in situ* hybridization on cryosections was carried out as previously described (Kaltezioti et al., 2010; Kaltezioti et al., 2014). The RNA probes complementary to *Lacuna* were prepared and labeled with digoxigenin.

### Statistical Analysis

All experimental designs are explained in each part of the section “materials and methods,” respectively. The normal distribution of values was verified with the Shapiro–Wilk normality test using IBM SPSS Statistics for Windows, Version 20.0. To ensure the reproducibility of results, all experiments were performed independently three to four times as indicated in each figure legend. For statistical analysis all measurements and experimental values from independent experiments were estimated with two-tailed Student’s t-test or two-way ANOVA. All the results are shown as mean ± SD. The exact *p* values are described in each figure legend, *p* values <0.05 are considered statistically significant. All analyses were done using Microsoft Excel 2013 and GraphPad Prism 8.

## Results

### *Lacuna* lncRNA Is Expressed in the Developing Murine Cortex

**FIGURE 1:**
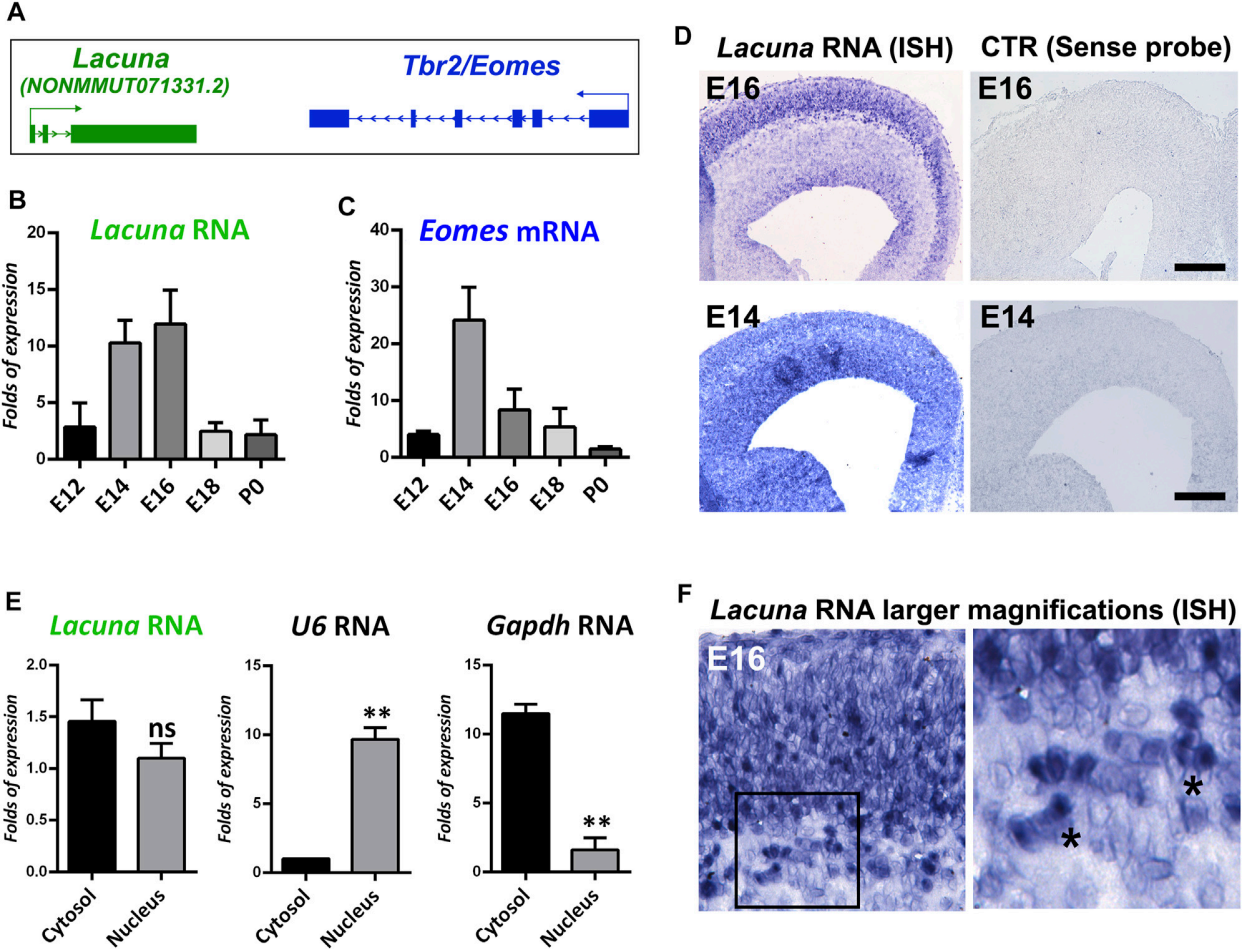
*Lacuna* is expressed in the mouse brain during embryonic development. **(A)** Schematic representation of *Lacuna* and Tbr2/Eomes locus. **(B)** RNA levels of *Lacuna* during mouse embryonic brain development. **(C)** mRNA levels of Eomes during mouse embryonic brain development. **(D)** *In situ* hybridization of *Lacuna* in cryosections from E16 and E14 mouse embryonic brain with the corresponding controls, as indicated. **(E)** Subcellular fractionation of NSCs and RNA levels of *Lacuna* in each subcellular compartment. mRNA levels of U6 and Gapdh were used to verify the fractionation of cells (**p < 0.01, *n* = 3). **(F)** Higher magnification micrographs of the *In situ* hybridization experiment with *Lacuna* specific riboprobe in E16 mouse embryonic brain **(left panel)**. Image in the right panel depicts larger magnification (40x) of the area included in the square shape of the image in the left panel (20x). Asterisks indicate representative cells where the *Lacuna* is localized both in cytosol and nucleus.

We and others had previously reported that a number of lncRNA genes are found in close genomic proximity (less than a 2 kb distance) to genes encoding for transcription factors with critical regulatory roles in neural development (Antoniou et al., 2014; Ponjavic et al., 2009). We hypothesized that a subset of these lncRNAs may be also involved in neural development by directly affecting the expression of neighboring protein coding genes. Thus, we decided to focus on such a pair of transcription factor/lncRNA genes, and more specifically on the *Lacuna* lncRNA, which is 1,661 nt long (sequence information in Supplementary Figure S1A) and transcribed from a genomic locus, only 1.5 kb far from the Eomes gene (Figure 1A). *Lacuna* has not been previously studied or reported in the literature, the only reference for this transcript is its presence in the RNA-seq databases from large scale consortia, where it is catalogued as TCONS_00034309 or NONMMUT071331.2 (NONCODE database). We have renamed this transcript and corresponding gene as *Lacuna*. RNA-seq data from NONCODE suggest expression in various adult mouse tissues, including heart, liver, lung, spleen, thymus, and hippocampus (Supplementary Figure S1B). On the other hand, the protein coding gene of the pair, the Eomes gene, encodes a transcription factor with a well-established role in promoting neuronal differentiation in cortical development (Arnold et al., 2008b; Vasistha et al., 2015; Mihalas et al., 2016; Sessa et al., 2017; Hevner, 2019). An intriguing question arising from these observations is whether *Lacuna* is playing any role in neural development either *via* an *in cis* effect on Eomes gene expression or an independent function.

To tackle this question, we initially investigated the expression pattern of *Lacuna* in the murine cortex during development. In particular, by real time RT-qPCR assays, we showed that *Lacuna* is differentially expressed during cortical development, with its peak of expression to be observed in the time window between E14 and E16, and then declines dramatically in E18 and P0 (Figure 1B). Its expression pattern is similar to the expression pattern of the neighboring Eomes gene (Figures 1B,C), although a shift towards later developmental stages is also evident in the case of *Lacuna*. As *Lacuna* is not yet annotated, we wanted to verify its RNA sequence and its exon-to-exon junctions, as reposited in the NONCODE database (Supplementary Figure S1A). To do that, we designed multiple sets of primers specific for the exon-exon boundaries as well as for amplicons including a combination of exons in order to verify the reposited sequence (Supplementary Figure S2A). We used these primers in RT-PCR reactions using as a starting material total cortical RNA from different developmental stages. All RT-PCR reactions produced products compatible with the sequence of *Lacuna* as reposited in NONCODE database (Supplementary Figure S2A). In addition, we showed that *Lacuna* is not subjected to alternative splicing in mouse developing cortex, as there are the expected exon-to-exon junctions and none of the exons is skipped during splicing (Supplementary Figure S2B). Next, *in situ* hybridization experiments confirmed the expression of *Lacuna* in the E16 and E14 murine cortex (Figure 1D), indicating a pattern of expression spanning the VZ/SVZ as well as outer cortical layers.

Furthermore, to define the subcellular localization of *Lacuna*, we performed subcellular fractionation of NSCs in conjunction with real time RT-qPCR. These NSCs were isolated from murine cortex at E14 and cultured *ex vivo*. Accordingly, we were able to show that *Lacuna* is localized both in the cytosol and the nucleus (Figure 1E). Control reactions for U6 RNA and Gapdh mRNA indicate that our nuclear and cytosolic fractions, respectively, were efficiently separated, further confirming our observation for *Lacuna*. In agreement, analysis of higher magnification images (20x and 40x) from the *in situ* hybridization experiments on E16 mouse embryonic cortex nicely corroborated these data, by showing distribution of *Lacuna* in both cellular compartments (Figure 1F). Therefore, *Lacuna* RNA is found both at cytoplasm and nucleus, indicating that this molecule may exert nuclear and/or cytoplasmic roles.

### *Lacuna* Overexpression Inhibits Neuronal Differentiation of NSCs

**FIGURE 2:**
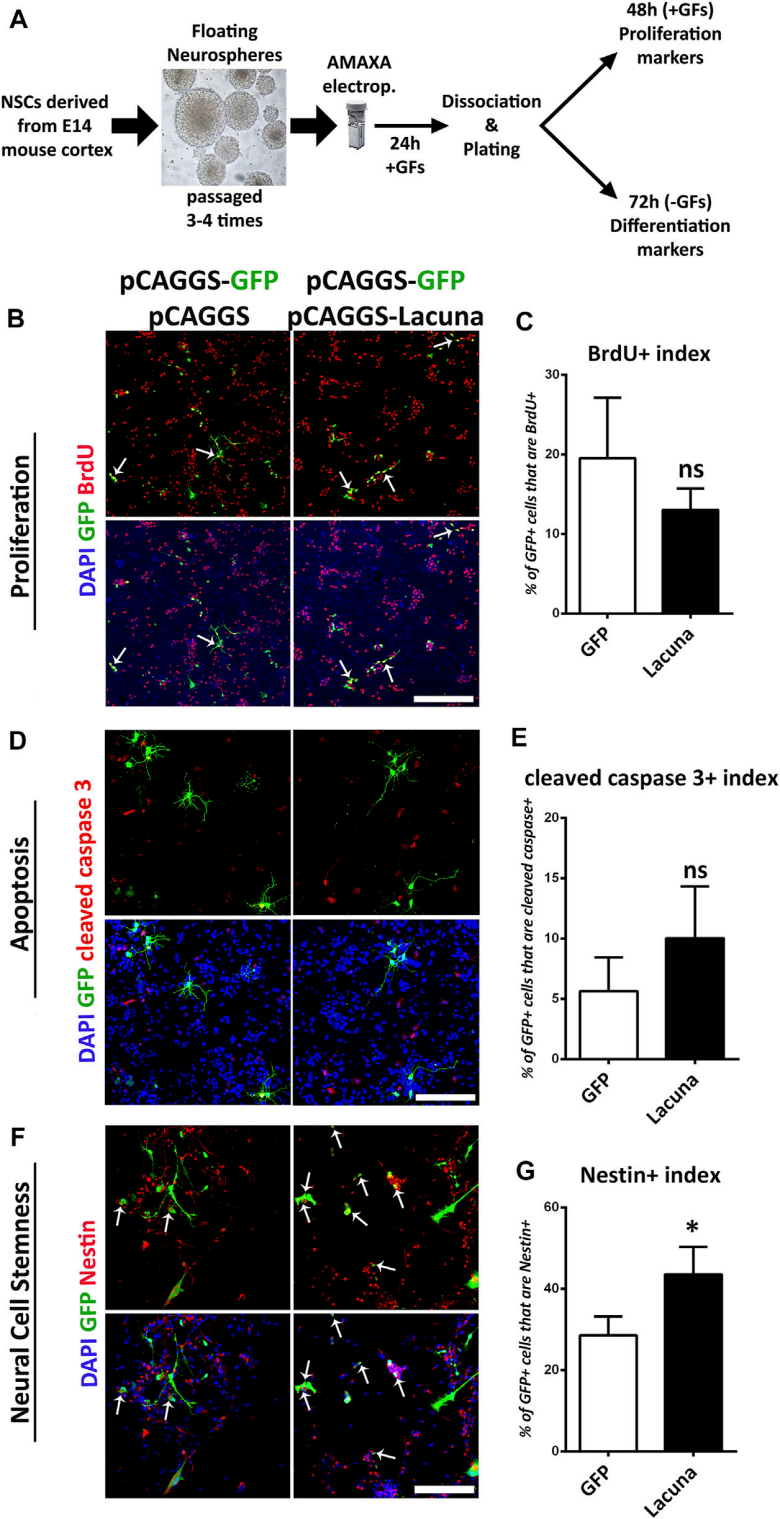
*Lacuna* overexpression affects stemness but not proliferation nor apoptosis of mouse Neural Stem Cells. **(A)** Schematic representation of our experimental strategy. NSCs are derived from E14 mouse cortices and they are cultured appropriately to form neurospheres over 3–4 passages. Neurospheres are transfected with plasmids of choice and then, they are dissociated and plated. In the presence of growth factors, NSCs proliferate, whereas without growth factors, they differentiate to generate neurons and astrocytes. **(B)** *Lacuna* and Control transfected Neural Stem cells were treated with BrdU for 2 h and then fixed and stained with anti-BrdU antibody (red), anti-GFP antibody (green) and 4, 6-diamidino-2-phenylindole (DAPI). Arrows indicate BrdU/GFP double positive cells. Scale bar: 250 μm. **(C)** Quantification of BrdU incorporation in transgene positive mouse Neural Stem cells (GFP: 19.55 ± 3.104%, *Lacuna*: 13.06 ± 1.098%, *p* > 0.05, *N* = 6 independent experiments, in total 596 cells for Control condition and 388 cells for *Lacuna* condition). **(D)** *Lacuna* and Control transfected mouse Neural Stem cells were immunostained for cleaved caspase 3 (red), and GFP (green) and labeled with DAPI. Scale bar: 250 μm. **(E)** Quantification of cleaved caspase 3 positive cells in transgene positive mouse Neural Stem cells (GFP: 5.629 ± 1.409%, *Lacuna*: 10.05 ± 2.144%, *p* > 0.05, *N* = 4 independent experiments, in total 374 cells for Control condition and 521 cells for *Lacuna* condition). **(F)** *Lacuna* and Control transfected mouse Neural Stem cells were immunostained for Nestin (red), GFP (green) and labeled with DAPI. Arrows indicate Nestin/GFP double positive cells. Scale bar: 100 μm. **(G)** Quantification of Nestin positive cells in transgene positive mouse Neural Stem cells (GFP: 28.59 ± 2.323%, *Lacuna*: 43.55 ± 3,378%, *p* < 0.05, *N* = 4 independent experiments, in total 363 cells for Control condition and 410 cells for *Lacuna* condition). For all cases, *p < 0.05, **p < 0.01, ***p < 0.001.

To gain insights into the functional role of *Lacuna* in neurodevelopment, we first studied the effect of its overexpression on *ex vivo* cultured NSCs, isolated from E14 murine cortex. Specifically, we constructed plasmids that were able to efficiently overexpress *Lacuna* and GFP under the CAGG promoter, which works well with mammalian cells as we have previously reported (Kaltezioti et al., 2021; Kaltezioti et al., 2010; Stergiopoulos and Politis, 2016). A mixture of two plasmids, pCAGGs-*Lacuna* and pCAGGs-GFP (experimental condition) or pCAGGS empty and pCAGGS-GFP (control condition), was used to transfect NSCs with Amaxa electroporation technique (Figure 2A). By this transfection strategy, we have previously shown that all cells marked with GFP are also transfected with the transgene (e.g., *Lacuna*) (Kaltezioti et al., 2010; Kaltezioti et al., 2014; Stergiopoulos and Politis, 2016). We have also confirmed this observation here (Supplementary Figure S3). In addition, by using Amaxa electroporation system, we have previously established and reported methodologies to perform gain- and loss-of-function experiments in embryonic NSCs as well as to extensively investigate the contribution of genes and molecular players in proliferation, differentiation, specification, and maturation of neural cells (Kaltezioti et al., 2010; Kaltezioti et al., 2014; Stergiopoulos and Politis, 2016), as also schematically described in Figure 2A.

**FIGURE 3:**
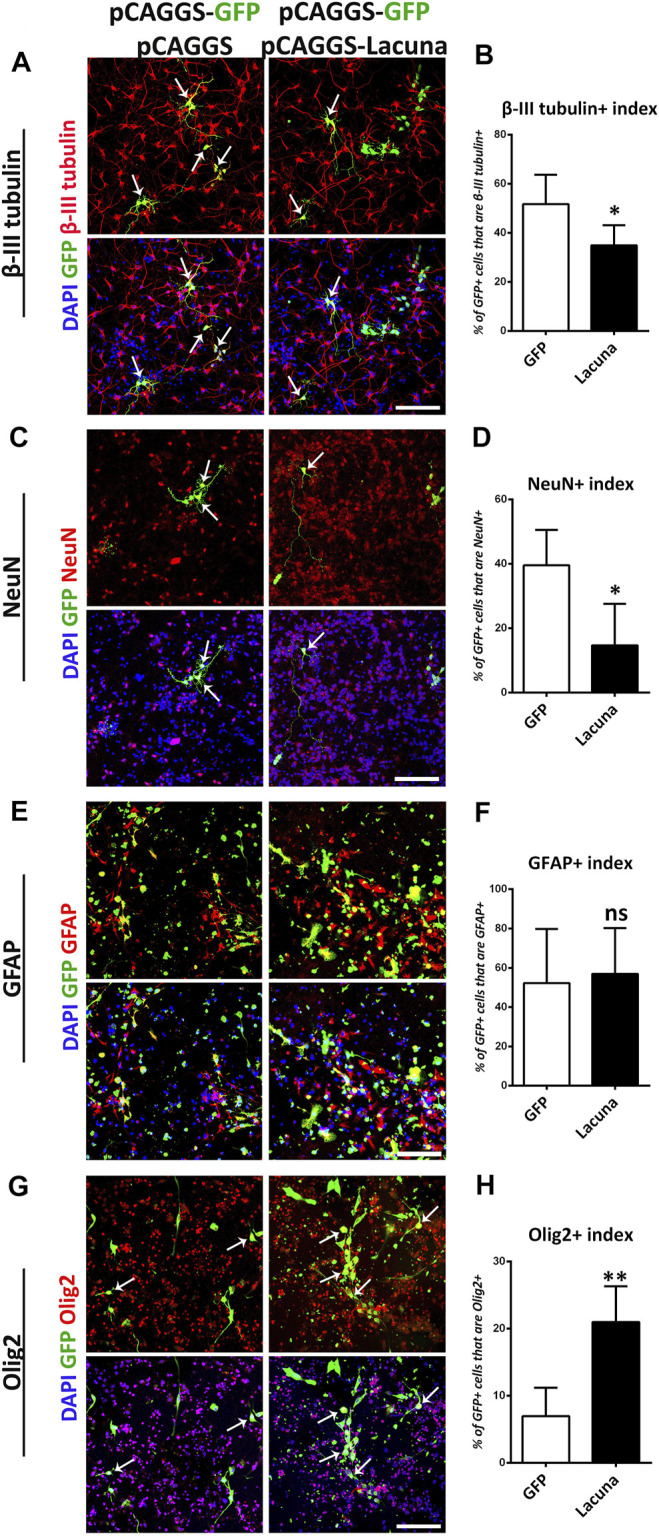
*Lacuna* overexpression inhibits neuronal differentiation of mouse Neural Stem Cells. **(A)** *Lacuna* and Control transfected mouse Neural Stem cells were immunostained for β-III tubulin (red) and GFP (green) and labeled with DAPI. Arrows indicate β-III tubulin/GFP double positive cells. Scale bar: 100 μm. **(B)** Quantification of β-III tubulin positive cells in transgene positive mouse Neural Stem cells (GFP: 51.73 ± 5.355%, *Lacuna*: 34.91 ± 3.671%, *p* < 0.05, *N* = 5 independent experiments, in total 498 cells for Control condition and 602 cells for *Lacuna* condition). **(C)** *Lacuna* and Control transfected mouse Neural Stem cells were immunostained for NeuN (red), GFP (green) and labeled with DAPI. Arrows indicate NeuN/GFP double positive cells. Scale bar: 100 μm. **(D)** Quantification of NeuN positive cells in transgene positive mouse Neural Stem cells (GFP: 39.58 ± 4.913%, *Lacuna*: 14.74 ± 6.426%, *p* < 0.01, *N* = 5 independent experiments, in total 280 cells for Control condition and 227 cells for *Lacuna* condition). **(E)** *Lacuna* and Control transfected mouse Neural Stem cells were immunostained for GFAP (red), GFP (green) and labeled with DAPI. Scale bar: 100 μm. **(F)** Quantification of GFAP positive cells in transgene positive mouse Neural Stem cells (GFP: 52.25 ± 13.76%, *Lacuna*: 56.94 ± 11.68%, *p* > 0.05, *N* = 4 independent experiments, in total 879 cells for Control condition and 468 cells for *Lacuna* condition). **(G)** *Lacuna* and Control transfected mouse Neural Stem cells were immunostained for Olig2 (red), GFP (green) and labeled with DAPI. Arrows indicate Olig2/GFP double positive cells. Scale bar: 100 μm. **(H)** Quantification of Olig2 positive cells in transgene positive mouse Neural Stem cells (GFP: 6.961 ± 1.905%, *Lacuna*: 21.00 ± 2.387%, *p* < 0.05, *N* = 5 independent experiments, in total 374 cells for Control condition and 521 cells for *Lacuna* condition). For all cases, *p < 0.05, **p < 0.01, ***p < 0.001.

Accordingly, we found that *Lacuna* overexpression is not affecting proliferation or apoptosis of *ex vivo* cultured NSCs (Figures 2B–E), yet it is sufficient to significantly induce the numbers of Nestin+ cells, a marker of neural cell stemness (Figures 2F,G). However, by using Sox2, another neural stem cell marker, we were not able to detect any statistically significant differences in the numbers of the Sox2+ cells after *Lacuna* overexpression as compared to control condition, although a trend towards an increase is evident (Supplementary Figures S4A,B). These data probably suggest that only a specific subset of progenitor cells is affected by this lncRNA. Remarkably, *Lacuna* overexpression caused a significant reduction in the ability of NSCs to produce βΙΙΙ-tubulin+ neurons (Figures 3A,B) and NeuN + neurons (Figures 3C,D) under differentiation conditions [without growth factors (GF)] as compared to the control condition. To exclude the possibility of a non-specific effect due to our electroporation strategy (e.g., due to unequal distribution of our tracing GFP plasmid), we tested whether an unrelated lncRNA is able to reproduce the same phenotype (Supplementary Figure S5). This unrelated lncRNA was not sufficient to reproduce the effect of *Lacuna* on neuronal differentiation, indicating a specific action. Most interestingly, astrogliogenic differentiation (GFAP marker) remains unaffected under *Lacuna* overexpression (Figures 3E,F). However, we found a *Lacuna*-mediated increase in the population of Olig2+ cells (Figures 3G,H). We assume that this extra population corresponds to Olig2-expressing neural progenitor cells that are not able to differentiate into neurons. In agreement, the differentiation into oligodendrocyte lineage is not affected, as shown by using the O4 oligodendrocyte progenitor marker (Supplementary Figures S4C,D). Therefore, these observations suggest that *Lacuna* is sufficient to exert a significant effect on the ability of NSCs to differentiate into neurons without affecting gliogenic or proliferative capacities of these cells.

### *Lacuna* Knockdown Promotes Differentiation of NSCs

**FIGURE 4:**
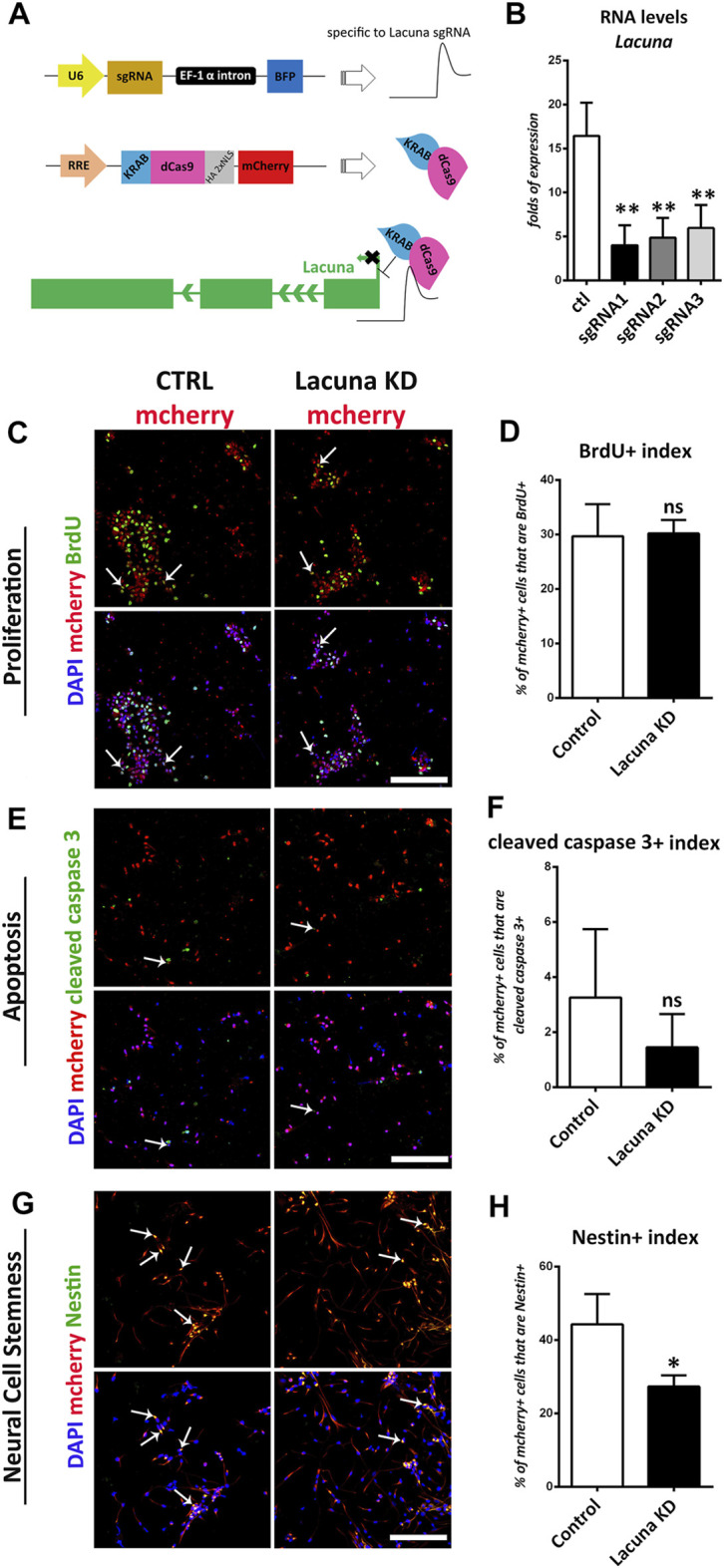
*Lacuna* knockdown reduces stemness but does not affect proliferation nor apoptosis of mouse Neural Stem Cells. **(A)** Scheme of dCas9-KRAB effector system and *Lacuna* knockdown strategy. The first plasmid expresses the guide RNAs that target *Lacuna*, the second plasmid expresses dCas9-KRAB and mcherry. When transfected together in Neural Stem cells, guide RNA recruits dCas9-KRAB fusion protein to *Lacuna* and inhibits its expression. In control cultures, NSCs were transfected with both plasmids, but first plasmid lacked a guide RNA sequence. **(B)** Three different guide RNA sequences were used to target *Lacuna* gene. All constructs were efficient in knocking down *Lacuna* expression. We selected sgRNA1 to proceed further. **(C)** Mouse Neural Stem cells were transfected with dCas9-KRAB-mcherry and sgRNA1 targeting *Lacuna* (*Lacuna* KD-mcherry) or no guide RNA (CTRL-mcherry). They were treated with BrdU for 2 h and then fixed and stained with anti-BrdU antibody (green), anti-mcherry (red) and 4, 6-diamidino-2-phenylindole (DAPI). Scale bar: 100 μm. **(D)** Quantification of BrdU incorporation in dCas9-KRAB-mcherry positive mouse Neural Stem cells (Control: 29.67 ± 2.63%, *Lacuna* KD: 30.18 ± 1.115%, *p* > 0.05, *N* = 5 independent experiments, in total 369 cells for Control condition and 469 cells for *Lacuna* condition). **(E)** Mouse Neural Stem cells were transfected with dCas9-KRAB-mcherry and sgRNA1 targeting *Lacuna* (*Lacuna* KD-mcherry) or no guide RNA (CTRL-mcherry). They were immunostained for cleaved caspase 3 (green), mcherry (red) and labeled with DAPI. Scale bar: 100 μm. **(F)** Quantification of cleaved caspase 3 positive cells in dCas9-KRAB-mcherry positive mouse Neural Stem cells (Control: 3.261 ± 1.013%, *Lacuna* KD: 1.448 ± 0.4918%, *p* > 0.05, *N* = 6 independent experiments, in total 644 cells for Control condition and 1,261 cells for *Lacuna* condition) **(G)** Mouse Neural Stem cells were transfected with dCas9-KRAB-mcherry and sgRNA1 targeting *Lacuna* (*Lacuna* KD-mcherry) or no guide RNA (CTRL-mcherry). They were immunostained for Nestin (green), mcherry (red) and labeled with DAPI. Scale bar: 100 μm. **(H)** Quantification of Nestin positive cells in dCas9-KRAB-mcherry positive mouse Neural Stem cells (Control: 44.24 ± 4.806%, *Lacuna* KD: 27.31 ± 1.752%, *p* < 0.05, *N* = 3 independent experiments, in total 408 cells for Control condition and 512 cells for *Lacuna* condition). For all cases, *p < 0.05, **p < 0.01, ***p < 0.001.

To further investigate the involvement of *Lacuna* in NSCs fate decision, we assessed whether it is necessary for NSCs differentiation by performing knockdown experiments using a CRISPR-dCas9-KRAB effector system (Alerasool et al., 2020; Parsi et al., 2017). This system is highly effective and specific in knocking down lncRNAs expression, but at the same time it leaves DNA intact, meaning that there are no changes at the level of DNA sequence (Figure 4A), as is the case with the traditional CRISPR-Cas9 methodology. This feature is extremely helpful in the case of lncRNAs research, since it has been previously shown that deletion of lncRNAs genomic loci may lead to significant effects on cellular functions due to the DNA changes (e.g., deletion of regulatory DNA elements) and not due to the downregulation of lncRNA expression (Paralkar et al., 2016; Kopp and Mendell, 2018). To achieve the CRISPR-dCas9-KRAB-mediated knockdown of *Lacuna* RNA expression, we utilized 3 different guide RNAs (sgRNAs). All of them have been designed in such a way (GenCRISPR gRNA Design Tool) to target the first exon of *Lacuna* gene. Thus, we showed that all three of them are able to downregulate the expression of *Lacuna* RNA (Figure 4B), so we continued our studies with the gRNA that had the strongest effect.

**FIGURE 5:**
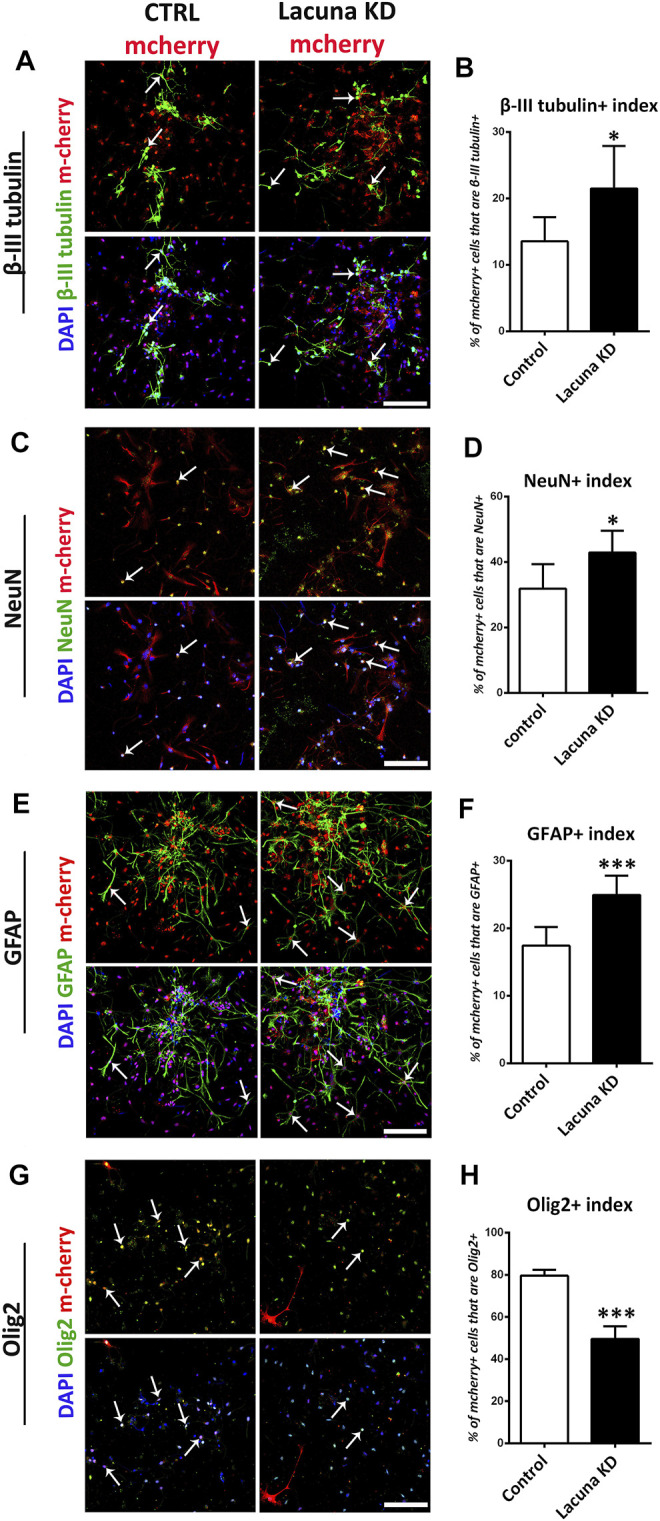
*Lacuna* knockdown promotes differentiation of mouse Neural Stem Cells. **(A)** Mouse Neural Stem cells were transfected with dCas9-KRAB-mcherry and sgRNA1 targeting *Lacuna* (*Lacuna* KD-mcherry) or no guide RNA (CTRL-mcherry). They were immunostained for β-III tubulin (green), mcherry (red) and labeled with DAPI. Scale bar: 100 μm. **(B)** Quantification of β-III tubulin positive cells in dCas9-KRAB-mcherry positive mouse Neural Stem cells (Control: 13.54 ± 1.481%, *Lacuna* KD: 21.50 ± 2.617%, *p* < 0.05, *N* = 6 independent experiments, in total 1,047 cells for Control condition and 1,438 cells for *Lacuna* condition). **(C)** Mouse Neural Stem cells were transfected with dCas9-KRAB-mcherry and sgRNA1 targeting *Lacuna* (*Lacuna* KD-mcherry) or no guide RNA (CTRL-mcherry). They were immunostained for NeuN (green), mcherry (red) and labeled with DAPI. Scale bar: 100 μm. **(D)** Quantification of NeuN positive cells in dCas9-KRAB-mcherry positive mouse Neural Stem cells (Control: 31.86 ± 3.062%, *Lacuna* KD: 42.86 ± 2.723, *p* < 0.05, *N* = 6 independent experiments, in total 89 cells for Control condition and 81 cells for *Lacuna* condition) **(E)** Mouse Neural Stem cells were transfected with dCas9-KRAB-mcherry and sgRNA1 targeting *Lacuna* (*Lacuna* KD-mcherry) or no guide RNA (CTRL-mcherry). They were immunostained for GFAP (green), mcherry (red) and labeled with DAPI. Arrows indicate GFAP/mcherry double positive cells. Scale bar: 100 μm. **(F)** Quantification of GFAP positive cells in dCas9-KRAB-mcherry positive mouse Neural Stem cells (Control: 17.43 ± 1.124%, *Lacuna* KD: 24.94 ± 1.165%, *p* < 0.001, *N* = 6 independent experiments, in total 679 cells for Control condition and 543 cells for *Lacuna* condition) **(G)** Mouse Neural Stem cells were transfected with dCas9-KRAB-mcherry and sgRNA1 targeting *Lacuna* (*Lacuna* KD-mcherry) or no guide RNA (CTRL-mcherry). They were immunostained for Olig2 (green), mcherry (red) and labeled with DAPI. Scale bar: 100 μm. **(H)** Quantification of Olig2 positive cells in dCas9-KRAB-mcherry positive mouse Neural Stem cells (Control: 79.59 ± 1.394%, *Lacuna* KD: 49.53 ± 3.023%, *p* < 0.001, *N* = 4 independent experiments, in total 584 cells for Control condition and 306 cells for *Lacuna* condition). For all cases, *p < 0.05, **p < 0.01, ***p < 0.001.

In good agreement with the overexpression studies, proliferation and apoptosis are not affected by *Lacuna* knockdown in NSCs (Figures 4C–F), while the number of Nestin+ cells is significantly decreased (Figures 4G,H). Similar to the overexpression experiments, *Lacuna* knockdown is not affecting the numbers of Sox2+ cells (Supplementary Figures S6A,B). On the other hand and conversely to the overexpression studies, *Lacuna* knockdown in primary NSCs resulted in a significant increase of β-III tubulin+ neurons (Figures 5A,B) and NeuN+ neurons (Figures 5C,D), but also of GFAP+ astrocytes (Figures 5E,F), as shown by immunofluorescent experiments in the absence of GFs. The Olig2+ population was found decreased (Figures 5G,H), hence exhibiting an opposite effect than that of *Lacuna* overexpression condition. Likewise, the numbers of O4+ cells, marking the oligodendrocyte lineage, are not affected (Supplementary Figures S6C,D). Taken together, these observations indicate that *Lacuna* RNA is critically involved in the regulation of neuronal differentiation in NSCs.

### *Lacuna* Is Necessary for Eomes Expression in NSCs

**FIGURE 6:**
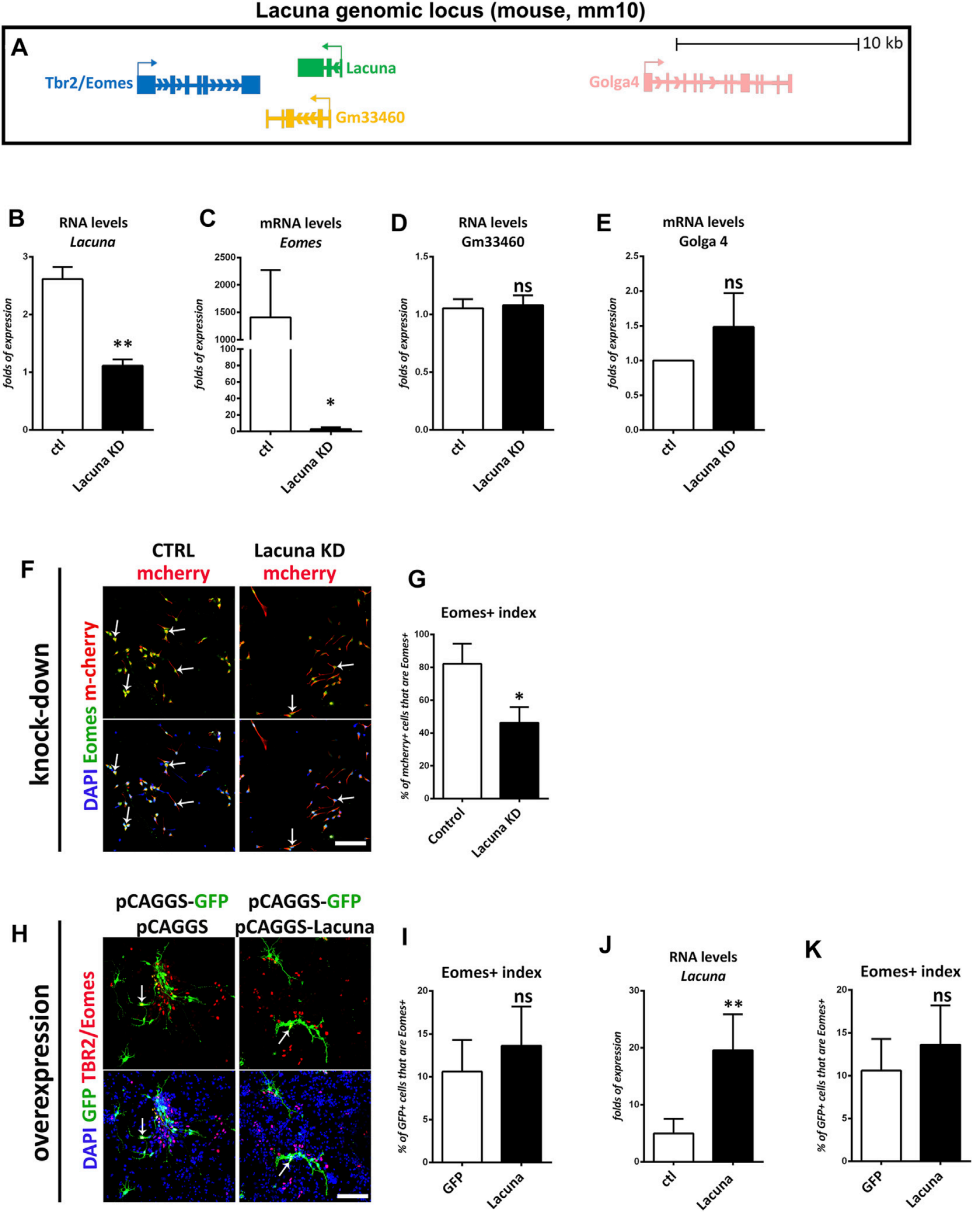
*Lacuna* is necessary for Eomes expression in mouse Neural Stem Cells. **(A)** Scheme of *Lacuna* and Eomes locus on mouse chromosome 9. Despite their vicinity, Gm33460 and Golga4 are not affected by guide RNAs targeting *Lacuna*. **(B)** RNA levels of lncRNA *Lacuna* upon *Lacuna* knockdown **(C)** mRNA levels of Eomes upon *Lacuna* knockdown **(D)** RNA levels of Gm33460 upon *Lacuna* knockdown **(E)** mRNA levels of Golga4 upon *Lacuna* knockdown **(F)** Mouse Neural Stem cells were transfected with dCas9-KRAB-mcherry and sgRNA1 targeting *Lacuna* (*Lacuna* KD-mcherry) or no guide RNA (CTRL-mcherry). They were immunostained for Eomes (green), mcherry (red) and labeled with DAPI. Scale bar: 100 μm. **(G)** Quantification of Eomes positive cells in dCas9-KRAB-mcherry positive mouse Neural Stem cells (Control: 82.13 ± 7.091%, *Lacuna* KD: 46.27 ± 5.534%, *p* < 0.05, *N* = 3 independent experiments, in total 239 cells for Control condition and 281 cells for *Lacuna* condition). **(H)** *Lacuna* and Control transfected mouse Neural Stem cells were immunostained for Eomes (red), GFP (green) and labeled with DAPI. Scale bar: 100 μm. **(I)** Quantification of Eomes positive cells in transgene positive mouse Neural Stem cells (GFP: 10.60 ± 1.655%, *Lacuna*: 13.62 ± 2.046%, *p* > 0.05, *N* = 5 independent experiments, in total 383 cells for Control condition and 309 cells for *Lacuna* condition). **(J)** RNA levels of lncRNA *Lacuna* upon *Lacuna* overexpression **(K)** mRNA levels of Eomes upon *Lacuna* overexpression. For all cases, *p < 0.05, **p < 0.01, ***p < 0.001.

Next, we wanted to investigate whether the effect of *Lacuna* on NSCs is mediated through a possible action on the Eomes gene expression. Since it has been previously reported that Eomes facilitates neuronal differentiation, we would expect a negative action of *Lacuna* on Eomes expression. Towards this direction, we examined whether knockdown of *Lacuna* affects the mRNA expression of Eomes and other genes in its genomic neighborhood. First, we focused on the other genes of the locus to confirm the specificity of our approach. Accordingly, we searched for possible effects on neighboring to *Lacuna* genes and specifically, on Golga4 gene and a recently annotated non-coding RNA gene, Gm33460 (Figure 6A). Golga4 is approximately 16,500 bp away from the 5’ of *Lacuna* and it encodes one of the golgins, a family of proteins localized in the Golgi apparatus. Gm33460 is downstream to *Lacuna* with a small common sequence shared between these two transcripts (end of 2nd exon and beginning of 3rd exon), but it continues after the RNA sequence of *Lacuna* (Figure 6A). Notably, both Golga4 and Gm33460 are not affected by KRAB-dCas9 that is targeted to *Lacuna* sequence, as shown by real time RT-qPCR assays (Figures 6B–E). These observations suggest that our knockdown approach is specific and sufficient to downregulate *Lacuna* expression, without affecting the other two genes, which are found close to *Lacuna* transcription start site (TSS).

Surprisingly, upon knockdown of *Lacuna* in NSCs and under minus growth factor conditions, Eomes gene expression is downregulated (Figures 6B,C). Consistently, knockdown of *Lacuna* induces a statistically significant downregulation of the Eomes expression at the protein level as well, as shown with immunofluorescence experiments (Figures 6F,G). On the other hand, *Lacuna* overexpression does not affect the numbers of Eomes+ cells, nor Eomes gene expression at the mRNA level (Figures 6H–K). This difference between knockdown and overexpression experiments probably indicates that *Lacuna* is able to regulate Eomes gene only *in cis*. Thus, only when we are knocking down the cis-expressed *Lacuna* gene, we are observing an effect on Eomes gene expression. On the other way round, when we are providing *Lacuna* transcript *in trans*, by exogenously overexpressing it, we are not able to detect any effect on Eomes gene expression. Consequently, we favor a conclusion that *Lacuna* is positively regulating the expression of Eomes *in cis*.

However, this positive action cannot explain the effect of *Lacuna* on neuronal differentiation, since Eomes promotes neuronal differentiation (Englund et al., 2005; Arnold et al., 2008a; Arnold et al., 2008b; Sansom et al., 2009). Therefore, *Lacuna*-mediated regulation of Eomes gene expression cannot explain its role in inhibiting neuronal differentiation. In agreement, *Lacuna* overexpression can inhibit neuronal differentiation without affecting Eomes expression. Thus, we propose a hypothetical model where *Lacuna* exerts an Eomes-independent effect on differentiation *via* a mechanistic action in the nucleus and/or in the cytoplasm.

## Discussion

The complexity of the mammalian brain is mainly due to the huge numbers of neurons and glial cells that interact to form its underlying structure. All these cells are derived from a pool of neural stem cells that proliferate with enormous rates and then differentiate to generate first neurons and then glial cells. The differentiation of neural stem cells towards the neuronal or glial cell identity is a major developmental process controlled by the interplay between extracellular signaling cues and intrinsic gene regulation networks (Martynoga et al., 2012; Segklia et al., 2012; Gallo and Deneen, 2014; Guérout et al., 2014; Paridaen and Huttner, 2014; Urban and Guillemot, 2014; Okawa et al., 2016; Lalioti et al., 2019). Elucidation of the molecular mechanisms that control these networks could provide valuable information on how the mammalian brain is formed as well as useful insights into the involvement of new molecular players in nervous system diseases, disorders, and cancers. It has recently become evident that a large part of the non-coding genome is transcribed producing RNA molecules that are not translated into proteins, but they exhibit tissue and cell-type specific patterns of expression (Maeda et al., 2006; Djebali et al., 2012; Fatica and Bozzoni, 2014). Among them, lncRNAs represent a large part of the mammalian genes and according to some estimations larger than the part of protein-coding genes (Carninci et al., 2005; Hosseini et al., 2019). Intriguingly, the lncRNAs that are expressed in the mammalian brain are preferentially harbored by genomic loci in the vicinity of brain-specific, transcriptionally active during development, protein-coding genes (Ponjavic et al., 2009; Antoniou et al., 2014). Here we wanted to investigate the relationship between lncRNAs and protein-coding genes, therefore we decided to focus on the Eomes genomic locus. From this locus, the *Lacuna* lncRNA is transcribed in a close genomic proximity to the gene encoding for the transcription factor Eomes. The rationale for choosing Eomes-*Lacuna* genomic locus is the fact that Eomes is a key player in neuronal differentiation during cortical neurogenesis (Englund et al., 2005; Arnold et al., 2008b; Vasistha et al., 2015; Sessa et al., 2017; Hevner, 2019). On the other hand, almost nothing was known about *Lacuna* in the nervous system or other tissues or cell types. We showed here that *Lacuna* expression is significantly induced in the murine embryonic cortex at E14 and remains high until E16, to be then reduced at E18 and P0. *Lacuna* expression is similar to that of Eomes (Bulfone et al., 1999; Kimura et al., 1999; Englund et al., 2005; Vasistha et al., 2015), suggesting a common regulation of these two genes or a synergistic interaction between them. Indeed, with our knockdown strategy in primary NSC cultures, we revealed that *Lacuna* is necessary for Eomes gene expression. However, exogenous overexpression of *Lacuna* is not sufficient to upregulate or in any way affect Eomes expression in NSCs. These observations indicate that *Lacuna* transcript can regulate Eomes expression only *in cis*. The *in cis* action of *Lacuna* is also supported by the presence of *Lacuna* transcripts in the nucleus of NSCs, indicating a function related to the regulation of gene expression. *Lacuna* is also equally distributed between nucleus and cytoplasm suggesting that it has additional roles in the cytoplasmic compartment.

However, the detailed molecular mechanism *via* which *Lacuna* may contribute to the transcriptional regulation of Eomes gene *in cis* is not clear and is still an open question. *Lacuna* does not seem to have any significant homology or similarity to other mouse genes, yet the possibility of acting as an enhancer element *via* DNA—RNA interaction cannot be ruled out. Another possibility is that *Lacuna* may guide chromatin remodeling complexes or it may recruits transcription factors, cofactors and RNA polymerases to the Eomes locus affecting its expression. These are cellular functions known for other lncRNAs (e.g., HOTAIR and EVF-2 respectively) (reviewed in Gutschner and Diederichs, 2012) and may explain the *in cis* action of *Lacuna* as well.

Yet, based on our observations we cannot exclude an alternative hypothetical scenario where *Lacuna* promoter is directly affecting the activity of Eomes promoter in a positive manner. In this scenario, *Lacuna* proximal promoter could act as an enhancer of the Eomes gene and therefore *via* DNA looping it could contribute to the activation of Eomes gene. Thus, by our CRISPR-dCas9-KRAB approach, we may inactivate both promoters. Although it is a valid possibility, we consider it quite unlikely to specifically inactivate Eomes promoter by this approach without affecting the other two transcripts of the genomic locus, taking also into account that all these transcripts belong to the same TAD (Topologically associating domain).

Eomes is transiently expressed in the cortical progenitor cells during embryonic development to promote neuronal differentiation (Englund et al., 2005; Arnold et al., 2008b). Based on these data and the positive role of *Lacuna* in Eomes gene expression, it could be assumed that *Lacuna* may also promote neuronal differentiation of NSCs. Surprising enough, we show that *Lacuna* exerts exactly the opposite action by inhibiting neurogenesis (βΙΙΙ-tubulin and NeuN markers) and promoting a neural progenitor cell-like identity (Nestin and Olig2 markers). However, Sox2, another major neural stem cell marker (Pevny and Nicolis, 2010), remains unaffected. This finding together with the fact that Nestin is not expressed exclusively in NSCs, but persists in immature primary cortical neurons (Mignone et al., 2004; Bott et al., 2019), could indicate that *Lacuna* disrupts neuronal maturation, thus generating a progenitor cell population unable to properly differentiate. Together these unexpected findings suggest that *Lacuna* affects neuronal differentiation *via* an Eomes-independent (*in trans*) mechanistic function on other gene(s) or pathways. Consistent with this scenario, overexpression of *Lacuna* inhibits neuronal differentiation without influencing Eomes expression. Yet, how *Lacuna* suppresses neuronal differentiation is still an open question. To this end, it is tempting to speculate that this effect of *Lacuna* is mediated by promoting the expression of Olig2. In agreement with this hypothesis, neurogenesis defects in *Lacuna* overexpressing NSCs are accompanied by a striking increase in the Olig2+ cells. The exact opposite effect on the numbers of Olig2+ cells were observed in NSCs that were lacking *Lacuna*. In accordance, it has been reported that Olig2 overexpression in neural stem cells elicits neurogenesis defects (Liu et al., 2015) and that Olig2 has also anti-neuronal functions in different developmental stages and depending on its phosphorylation state (Sun et al., 2011). Moreover, it is known that Olig2 antagonizes Ngn2 and inhibits the premature expression of post-mitotic motor neuron genes holding progenitor cells in reserve for later differentiation (Lee et al., 2005). Altogether, these observations may indicate that *Lacuna* is involved in the regulation of neurogenesis, probably through an Olig2-mediated pathway. Mechanistically, it has been shown that some lncRNAs accommodate small ORFs that are translated into functional peptides (Zhang et al., 2021), a hypothesis that is, still valid for these *in trans* actions of *Lacuna*, as it is also detected in the cytoplasm. Alternatively, other possible mechanisms through which *Lacuna* affects neurogenesis might be miRNA sequestration and mRNA stability (*via* mRNA-lncRNA pairing) of targets that would be extremely interesting to identify.

Moreover, the fact that two genes from the same genomic locus are co-expressed with a similar pattern, yet they exert opposite roles, may indicate that positive and negative effectors of a cellular phenomenon are co-regulated to fine-tune the final outcome. Therefore, it could be that a pro-neurogenic factor, such as Pax6, may induce the transcriptional activation of the chromatin domain that includes both genes to primarily promote the expression of Eomes, which in turn enhances neurogenesis. Simultaneously, a second transcript is produced from the same activation event, the *Lacuna* lncRNA, which partially counteracts the pro-neurogenic function of Eomes, to fine-tune the number of neurons that are produced from a given pool of NSCs or alternatively to delay the depletion of NSC pool and maintain their differentiation potential for longer time periods. However, this hypothesis is mainly based on observations from our *ex vivo* culture system of murine NSCs. It would be extremely interesting to test whether the same regulatory and cellular differentiation effects are observed on an *in vivo* system.

Overall, this hypothetical scenario may point to a new emerging paradigm in genome science, where lncRNAs are co-regulated with protein coding genes with opposite functions to fine-tune the cellular action of the latter.

## Data Availability

The raw data supporting the conclusion of this article will be made available by the authors, without undue reservation.

## Supplementary Material

Supplementary Figure S1
*Lacuna (NONMMUT071331.2)* sequence and expression profile from the NONCODE database. **(A)** *Lacuna* transcript sequence as it was retrieved by the NONCODE Genome Database (http://www.noncode.org/show_rna.php?id=NONMMUT071331&version=2&utd=1#). Note that Lacuna corresponds to the transcript NONMMUT071331.2 from the NONCODE. **(B)** *Lacuna (NONMMUT071331.2)* was found in the NONCODE Genome Database to be expressed in various adult mouse tissues, including adult mouse hippocampus.

Supplementary Figure S2 Mapping of Lacuna locus by RT-PCR assays. **(A)** Schematic representation of the Lacuna gene organization (upper panel) in the mouse genome. The pairs of specific primers that were used to map different exons of *Lacuna* with PCR, are indicated with blue (forward primers) and red (reverse primers) arrows. In the lower panel, the sizes in bp of the expected PCR products are indicated. **(B)** PCR products of each pair were analyzed by agarose gel electrophoresis. PCR template was cDNA (random primers) produced by RNA extracted from mouse embryonic telencephalon of each developmental stage (E12, E14, E16, E18, P0). DNA Ladder: Quick Load Purple 100bp DNA Ladder.

Supplementary Figure S3 Our dual-plasmid overexpression strategy results in 100% co-localization of the products of these plasmids. **(A)** Schematic representation of the experimental co-expression strategy which shows the pCAGGs-GFP plasmid, as well as the pCAGGs-ds-Red plasmid. The ratio of transfection is 3 (pCAGGs-GFP plasmid) to 1 (ds-Red plasmid). **(B)** Mouse Neural Stem Cells were transfected with pCAGGS-GFP and pCAGGs-ds-Red plasmid and immunostained for GFP and ds-Red protein. Note that in all cases GFP positive cells are always ds-Red positive. Scale bar: 100 µm.

Supplementary Figure S4 Lacuna over-expression does not affect the numbers of Sox2 positive and O4 positive cells. **(A)** *Lacuna* and Control transfected mouse Neural Stem cells were immunostained for Sox2 (red) and GFP (green) and labeled with DAPI. Arrows indicate Sox2/ GFP double positive cells. Scale bar: 100 µm. **(B)** Quantification of Sox2 positive cells in transgene positive mouse Neural Stem cells (GFP: 60,57 ± 10,23%, Lacuna: 76,28 ± 9,043%, p > 0.05, N = 3 independent experiments, in total 117 cells for *Control* condition and 112 cells for Lacuna condition). **(C)** *Lacuna* and *Control* transfected mouse Neural Stem cells were immunostained for O4 (red), GFP (green) and labeled with DAPI. Arrows indicate O4/ GFP double positive cells. Scale bar: 100 µm. **(D)** Quantification of O4 positive cells in transgene positive mouse Neural Stem cells (GFP: 34,13 ± 3,526%, Lacuna: 50,83 ± 4,977, p > 0.05, N = 3 independent experiments, in total 68 cells for Control condition and 90 cells for Lacuna condition). For all cases, **p* < 0.05, ***p* < 0.01, ****p* < 0.001.

Supplementary Figure S5 Overexpression of unrelated lncRNA TCONS_00025370 does not affect neurogenesis. **(A)** *TCONS_00025370* and Control transfected mouse Neural Stem cells were immunostained for β-III tubulin (red) and GFP (green) and labeled with DAPI. Arrows indicate β-III tubulin/GFP double positive cells. Scale bar: 75 µm. **(B)** Quantification of β-III tubulin positive cells in transgene positive mouse Neural Stem cells (GFP: 26,48 ± 5,781%, TCONS_00025370: 24,16 ± 3,415%, p > 0.05, N = 3 independent experiments, in total 363 cells for *Control* condition and 465 cells for *Lacuna* condition). For all cases, **p* < 0.05, ***p* < 0.01, ****p* < 0.001.

Supplementary Figure S6
*Lacuna* knockdown does not affect the numbers of Sox2 positive and O4 positive cells. **(A)** Mouse Neural Stem cells were transfected with dCas9-KRAB-mcherry and sgRNA1 targeting Lacuna (Lacuna KD-mcherry) or no guide RNA (CTRL-mcherry). They were immunostained for Sox2 (green), mcherry (red) and labeled with DAPI. Arrows indicate Sox2/ mcherry double positive cells. Scale bar: 100 µm. **(B)** Quantification of Sox2 positive cells in dCas9-KRAB-mcherry positive mouse Neural Stem cells (Control: 57,17 ± 4,969%, Lacuna KD: 45,08 ± 10,24%, p > 0.05, N = 3 independent experiments, in total 95 cells for Control condition and 76 cells for Lacuna condition). **(C)** Mouse Neural Stem cells were transfected with dCas9-KRAB-mcherry and sgRNA1 targeting Lacuna (Lacuna KD-mcherry) or no guide RNA (CTRL-mcherry). They were immunostained for O4 (green), mcherry (red) and labeled with DAPI. Arrows indicate O4/ mcherry double positive cells. Scale bar: 100 µm. (D) Quantification of O4 positive cells in dCas9-KRAB-mcherry positive mouse Neural Stem cells (Control: 67,36 ± 7,986%, Lacuna KD: 50,38 ± 4,977 p > 0.05, N = 3 independent experiments, in total 103 cells for Control condition and 90 cells for Lacuna condition). For all cases, **p* < 0.05, ***p* < 0.01, ****p* < 0.001.
