# Supplementary figures and images for: Long Non-Coding RNA Lacuna Regulates Neuronal Differentiation of Neural Stem Cells During Brain Development
Source: Front Cell Dev Biol. 2021 Nov 24;9:726857. doi: 10.3389/fcell.2021.726857 (PMC8653915; doi:10.3389/fcell.2021.726857)

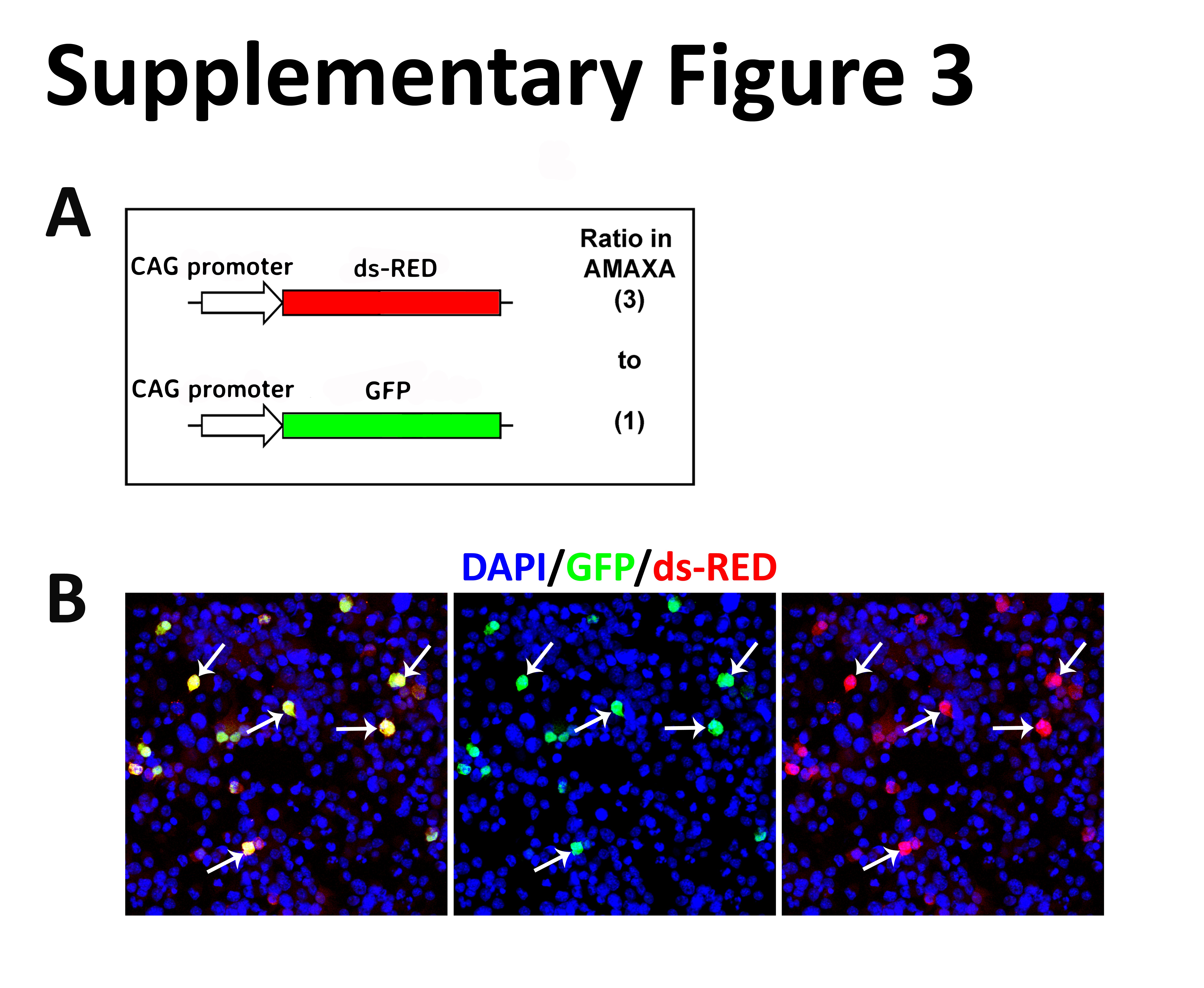

Supplement: Supplementary file 1 [file Image3.JPEG]

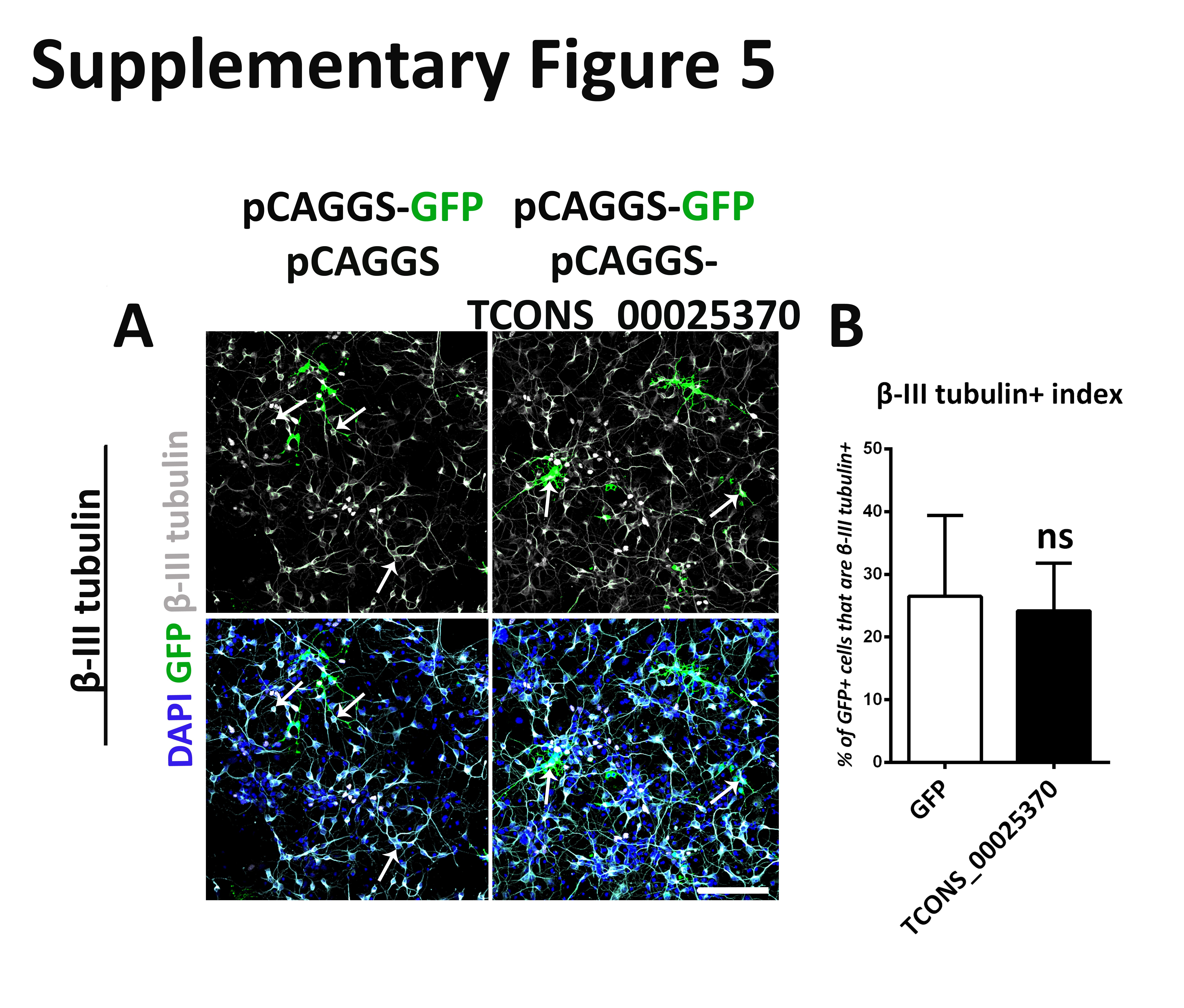

Supplement: Supplementary file 4 [file Image5.JPEG]

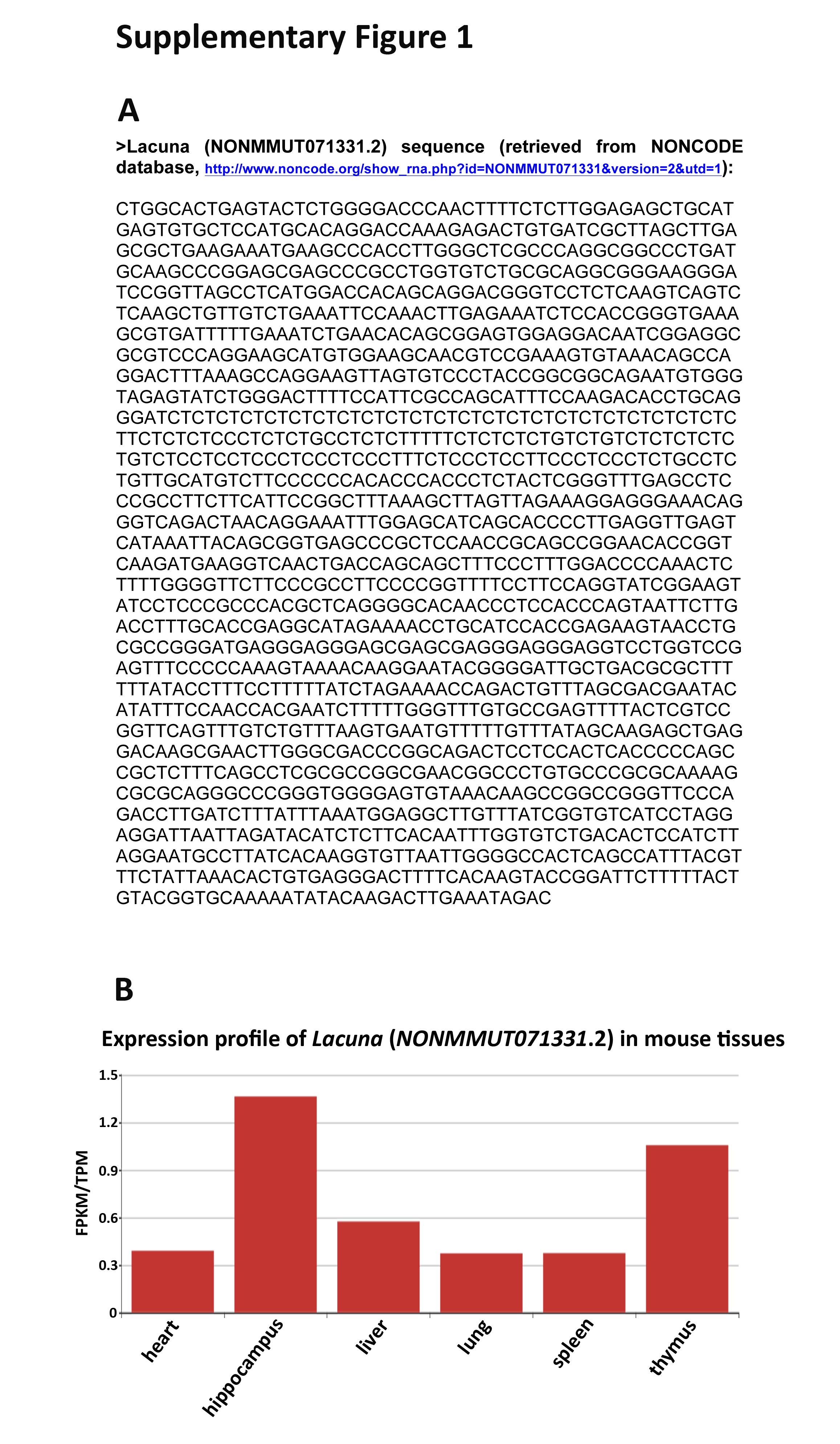

Supplement: Supplementary file 5 [file Image1.TIF]
